# What are the diagnoses attributed to persistent hip pain after hip arthroplasty? A systematic review

**DOI:** 10.1016/j.jcot.2025.103036

**Published:** 2025-05-09

**Authors:** Kael Hulin, Angie Fearon, Phil Newman

**Affiliations:** a University of Canberra Faculty of Health, Australia; b University of Canberra Research Institute for Sport and Exercise, Australia

**Keywords:** Hip replacement, Painfu l arthroplasty, Persistent Hip pain diagnositic algorithm

## Abstract

**Background:**

Persistent hip pain after total hip arthroplasty has been reported in up to 23 % of cases. Despite routine clinical tests, the source of pain often remains unclear, and diagnosis requires extensive investigation with imaging or surgical exploration.

**Aims:**

This systematic review aimed to identify the diagnoses attributable to the painful hip arthroplasty. The second aim was to identify diagnostic techniques used to identify them.

**Method:**

Three databases (Medline, Scopus and CINAHL) were searched from 2012 to 2024 using keywords and medical subject headings (MeSH) including ‘persistent pain AND hip arthroplasty AND diagnoses’. Quality assessment was undertaken with the Joanna Briggs Institute checklist for case-series. Data extraction was performed by one author using Covidence software and crosschecked by another for accuracy. Data included age, sex, arthroplasty type, diagnostic method, and confirmed diagnosis. Data was synthesised to provide a quantitative overview of diagnoses and diagnostic methods. PROSPERO number CRD42022340158.

**Results:**

The search and reference screening returned 285 papers of which seven high quality and two unclear quality case-series met the inclusion criteria. There was a total of 388 painful hip arthroplasties included. Prostheses loosening or infection was present in 28.6 % of cases. Iliopsoas impingement was present in 21 % of cases. Causes outside the hip accounted for 16.4 % of cases with referred pain from the lumbar spine (14.6 %) most common. Greater trochanteric pain syndrome (GTPS) was present in 13 %. The painful etiology remained unknown in 9.2 % of participants. The most common diagnostic imaging technique was x-ray (100 %) followed by magnetic resonance imaging (22 %).

**Conclusion:**

Prosthesis loosening and infection remain a significant cause of pain despite preliminary screening to exclude them. Iliopsoas impingement and causes outside the hip require significant consideration due to high prevalence. Less common differential diagnoses have been identified. X-ray is an important front-line imaging tool while other advanced imaging is used selectively to identify a diagnosis.

## 1 Introduction

Total hip arthroplasty (THA) is a routine medical intervention with many high-quality reports documenting success in reducing pain, restoring function, and improving quality of life.^1^, ^2^, ^3^ This treatment is one of the most common and successful major surgeries of this decade^4^^,^^5^ with a 20-year revision rate less than 3.5 %.^6^ Despite the marked success in improving function and quality of life, there is an increasing body of literature that documents persistent hip-pain after a hip-replacement.^4^^,^^7^^,^^8^ This has been termed ‘painful hip arthroplasty’ and is defined as persistent hip pain that outlasts the expected period of post-surgical pain^7^, typically three months.^9^ The prevalence of painful hip arthroplasties has been reported between 7 % and 23 % of participants.^4^ In 2020, almost 50,000 hip arthroplasties were performed in Australia, an increase of 52 % over the previous ten years.^1^^,^^6^ With the number of procedures predicted to increase in the next 30 years,^10^ more attention must be given to individuals who suffer from a painful hip arthroplasty.

Painful hip arthroplasty may be due to multiple, and sometimes difficult to determine etiologies. Identification may be done through techniques such as clinical examination, blood tests, imaging, anesthetic injection, arthroscopic investigation, and sometimes open revision surgery.^7^^,^^11^, ^12^, ^13^, ^14^, ^15^, ^16^, ^17^, ^18^, ^19^ Pain from prosthesis loosening and infection are the primary cause of revision surgery^6^^,^^20^^,^^21^ and are important to identify or rule out.^15^ Despite extensive clinical investigation and examination, alternative explanations for persistent pain may be difficult to establish with non-prosthetic related etiologies often poorly defined in medical literature.^7^ This lack of diagnostic clarity can increase the time to attain a diagnosis, delay treatment, and increase costs to insurers, individuals, and the health system.

There is no published consensus on the best method for diagnosing the cause of persistent pain after hip arthroplasty. The investigation is often done at the discretion of the attending clinician or local medical center guidelines.^12^^,^^15^^,^^21^ Existing diagnostic algorithms display poor consensus on diagnostic methods.^12^^,^^15^^,^^21^ Furthermore, there is limited research standardizing the approach to diagnose a painful hip arthroplasty. Thus, we undertook this systematic review to identify the known causes of painful hip-arthroplasty and report the diagnostic approach.

## 2 Aims and objective

This systematic review aimed to identify the diagnoses attributable to the painful hip arthroplasty. The second aim was to identify diagnostic techniques used to identify them.

## 3 Review protocol

The review protocol is registered on the international prospective register of systematic reviews (PROSPERO) and adheres to the PROSPERO guidelines for systematic reviews.^22^ The registration was completed on August 06, 2022, number CRD42022340158.

## 4 Research question

This review identifies the diagnoses attributed to persistent hip-pain after hip-arthroplasty. The research question was formulated using the CoCoPop framework, including condition, context, and population.^23^ The condition is persistent hip pain, and the context is the diagnoses that have been attributed to the condition. The population are participants with total hip arthroplasty.

## 5 Method

As pain due to loosening, infection or prosthesis failure have been well documented^24^, ^25^, ^26^, ^27^, ^28^, ^29^ studies limited in scope to loosening, infection or prosthesis failure were excluded from this review. Further, hip arthroplasty for this review is defined as total or revision hip arthroplasty. Persistent pain is defined as pain that lasts longer than three months after arthroplasty or pain that subsides and then returns. Hip pain is characterized by pain above the buttock crease and below the iliac crest in the anterior, lateral, posterior, or medial regions. A diagnosis is defined as the most likely cause attributed to the painful hip after systematic investigation. Nuances around the differential diagnosis of septic and aseptic loosening, infections, and prosthetic failure are beyond the scope of this review. These etiologies are reported together as “Prosthetic loosening or infection.” The search was completed with key words and Medical Subject Headings (MeSH) in the databases CINAHL, Medline via Web of Science, and Scopus. A search was performed in the Cochrane library with key terms (persistent hip pain AND hip arthroplasty AND diagnosis). These terms and synonyms of the terms were searched in the title and abstract of the above databases. A complete search strategy, including the synonyms, is in Appendix A. The search was limited to studies published from January 1, 2012 to November 28, 2024 to limit the findings to contemporary prosthesis etiologies. Studies not available in English were excluded. Search results were exported to EndNote (Version 20) and uploaded to Covidence (systematic review software from https://www.covidence.org) ^30^ for screening. The inclusion and exclusion criteria are presented in Table 1. This review was registered in PROSPERO number CRD42022340158.

**Table 1:** Eligibility criteria.

Inclusions	Exclusions
Population with total hip arthroplasty.	Revision total hip arthroplasty
Pain lasting three or more months after arthroplasty.	Pain reports within peri-operative recovery period <3 months
Published on or after January 1, 2012 until November 28, 2024	Combined data from hip with other joints. Unable to be differentiated.
Contains diagnoses or addresses etiology of painful hip arthroplasty.	Diagnosis coincides with another serious comorbidity.
Persistent pain that occurred after 3 months pain free post arthroplasty.	The article is about known infection or loosening of prosthesis only.
	Article is about risk factors.
	Article is about surgical technique.
	Study has less than five participants.

**Fig. 1:**
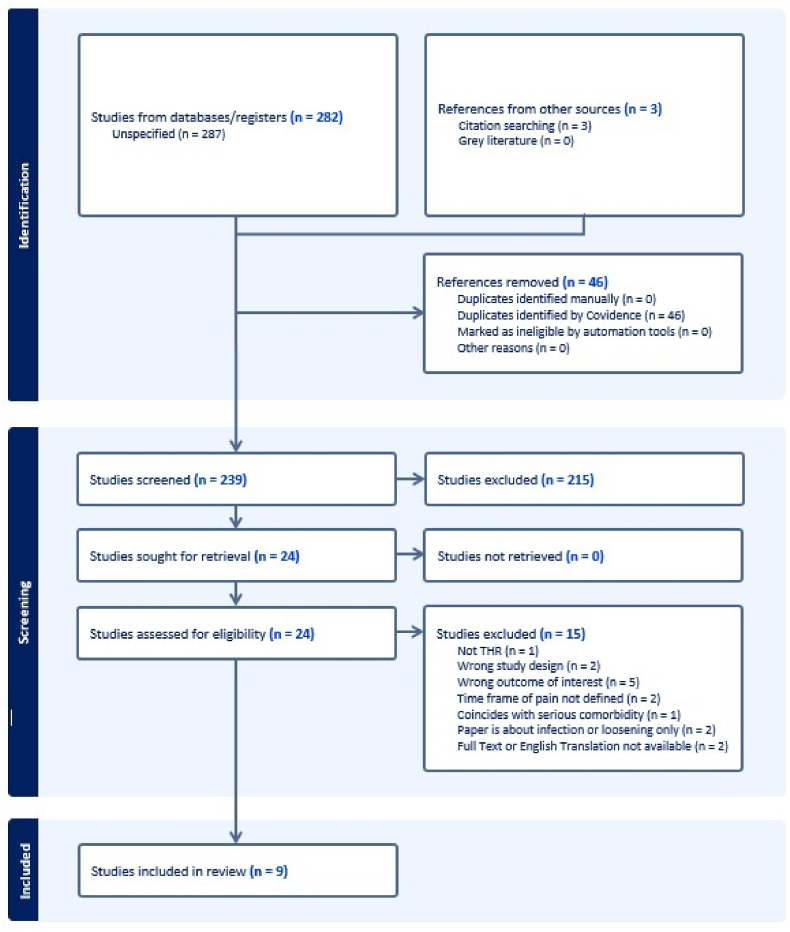
PRISMA flow diagram for paper screening and selection

Title and abstract screening were undertaken by K.H. and A.F. and conflicts settled by discussion. The full-text screening was undertaken by K.H. and P.N. and conflicts were settled by discussion. A manual search of the reference lists of screened systematic reviews was undertaken with an additional three articles identified for abstract screening. These were screened using the same method. The reasons for exclusion are stated in a Preferred Reporting Items for Systematic Reviews and Meta-Analyses (PRISMA) ^31^ flow diagram (Fig. 1).

Data extraction was completed by KH using Covidence software with one other researcher (AF) crosschecking 20 % of the extracted data ensuring accuracy. Data extracted included participant age, sex, type of arthroplasty, imaging or diagnostic methods, and diagnosis. For cases with multiple causes, the first or most likely was attributed to that case.

Quality assessment was done using the Joanna Briggs Institute (JBI) quality assessment checklist for case-series.^32^ Quality of studies was deemed ‘high’, ‘low’, or ‘unclear’ based on the following criteria. To be ‘high quality’ the study needs to meet all criteria of the appraisal tool. Studies of ‘unclear quality’ require one or more criteria to be unclear and all other criteria to be met. ‘Low quality’ studies would require one or more criteria not to be met. Answers that are ‘not applicable’ do not affect the quality of the study.

## 6 Results

The search and reference list screening returned 285 articles. Of these, 46 duplicates were removed by Covidence. Title and abstract screening removed 215 articles that were not relevant to the research question. The remaining 24 studies underwent full text review and a further 15 were excluded. Results of the screening are presented in the PRISMA^33^ flow diagram (Fig. 1). The remaining nine studies underwent data extraction and quality appraisal.

Appraisal with the JBI tool for case-series^32^ resulted in seven studies deemed of high quality and two of unclear quality (Table 2).

**Table 2:** Study summary.

Study author, year	Quality appraisal^32^	Study characteristics	Population characteristics			Diagnoses
			Mean age years (SD)	Males	Females	
Backer et al., 2020^11^	High quality	Prospective consecutive case-series with 37 painful THA were imaged with MRI and SPECT/CT. A single experienced surgeon made the diagnosis after clinical examination, plain radiography, MRI, and SPECT/CT.	61.8 (13.3)	15	22	15 Prosthetic loosening or infection.
						4 Iliopsoas impingement.
						4 GTPS.
						3 Undefined soft tissue pain.
						3 Gluteal and piriformis myalgia.
						2 Referred pain from the lumbar spine.
						1 Ischial tuberosity tendinitis.
						1 Pubic bone fracture.
						1 Painful pseudotumor.
						1 Iliopectinus bursitis.
						1 Ischiofemoral impingement.
						1 Scar tissue at abductor muscles.
Berber et al., 2015^12^	High quality	Observational imaging usefulness study on a prospective case-series of 19 metal-on-metal hip arthroplasties. Management after SPECT/CT was compared with management before SPECT/CT. Types of arthroplasties included 12 resurfacing arthroplasties, five revision arthroplasties, and two THA. Diagnosis was reached by consensus from 4 hip surgeons and 1 radiologist after clinical examination, CRP, plain radiography, blood metal ions, MARS MRI, and SPECT/CT.	51.7^10^	9	10	9 Unknown.
						5 Referred pain from lumbar spine.
						4 Infection or loosening.
						1 Non-union contralateral metatarsal fracture.
Chalmers et al., 2017^13^	High quality	Retrospective case-series of 49 participants with primary cementless THA treated for iliopsoas impingement. MoM and revision arthroplasties were excluded. Inclusion required normal CRP, ESR and plain radiograph, and positive active straight leg raise. Non-operative treatment of 20 participants included physiotherapy, NSAID's and iliopsoas tendon sheath corticosteroid injection. Operative treatment of 29 participants included acetabular revision (n = 21) or open iliopsoas tendon release (n = 8). Diagnosis was made based on symptoms, physical examination, and plain radiography, ± diagnostic tendon sheath injection.	64.8 (NR)	23	26	49 Iliopsoas impingement.
Dobrindt et al., 2015^14^	High quality	Consecutive retrospective case-series of 23 hips with persistent hip pain post arthroplasty. Inclusion required inconclusive plain radiograph of the hip, and normal CRP or ESR. All participants were imaged with bone scintigraphy, and SPECT/CT to detect the etiology. Diagnosis was made by consensus from experts in orthopaedic surgery, nuclear medicine, and radiology.	68.9^11^	16	7	7 Prosthetic loosening or infection.
						5 Referred pain from lumbar spine.
						5 Heterotopic ossification.
						5 Unknown.
						1 Neurological condition causing muscle imbalances.
Erivan et al., 2019^15^	High quality	Retrospective consecutive case-series with painful hips in 142 primary THA and 59 revision arthroplasties. Participants were excluded if they had a known cause of pain such as dislocation, fracture, or infection. Screening methods for these exclusions were not reported. The final diagnosis was attained by following a decision tree until confirmed. Notable tests included in the decision tree were CRP, ESR, WBC count, CT, ultrasonography, bone scintigraphy, plain radiography, MRI, and metal allergy tests.	56.5 (13.9)	79	122	83 Loosening, infection, or prosthesis failure.
						45 Referred pain from the lumbar spine.
						45 GTPS.
						7 Unknown.
						5 Iliopsoas impingement.
						3 Instability without dislocation.
						3 leg length discrepancy correction.
						3 Referred pain from the knee.
						2 Heterotopic ossification.
						1 Ischial tuberosity tendinitis.
						1 Fracture of the greater trochanter.
						1 Fracture of the ilioishiopubic ramus.
						1 Complex regional pain syndrome.
						1 Metabolic neuropathy.
Hart et al., 2012^16^	High quality	Case control study with 30 painful metal-on-metal THA were compared with 28 matched controls. Participants in the painful group were included if they had a painful MoM arthroplasty, unknown diagnosis after physical examination, negative blood tests for markers of infection, and normal radiographs. Diagnosis was attained after CT and MRI and later confirmed by revision surgery in some (n = 18) participants.	55 (13.3)	16	14	17 Pseudotumors.
						13 Unknown.
Lahner et al., 2013^17^	Unclear quality	Case-series of five primary THA participants with persistent hip pain six or more months after arthroplasty. Inclusion criteria required normal plain radiography, normal CRP, sterile hip aspiration, and unremarkable clinical examination. Two underwent CT imaging to exclude cup malposition. All underwent arthroscopy evaluation to reach diagnosis.	60.2 (4.3)	2	3	2 Prosthetic loosening or infection.
						2 Iliopsoas impingement.
						1 Adhesions of periprosthetic tissue.
Tassinari et al., 2021^18^	High quality	Consecutive retrospective case-series of 16 primary THA treated with arthroscopic iliopsoas tendon release. Diagnosis was confirmed with positive active straight leg raise, CT scan including 4th vertebrae and tibial plateau, and ultrasound guided iliopsoas peritendinous local anesthetic injection. Complete pain relief was achieved in 80 % of participants at > 2 year follow up.	57.8 (11.1)	5	11	14 Iliopsoas impingement.
						1 Heterotopic ossification.
						1 Femoral acetabular impingement
Yun et al., 2021^19^	Unclear quality	Retrospective case-series with eight THA participants who had failed conservative treatment for persistent hip pain. Fail treatment consisted of six months of physiotherapy, anti-inflammatories, and tendon sheath injection. The final diagnosis was reached after physical examination, plain radiography, CT imaging and diagnostic iliopsoas tendon sheath injection (one participants declined injection). All underwent open partial psoas tendon release with acetabular, cup or femoral head revision.	62 (12.3)	6	2	8 Iliopsoas impingement caused by cup malposition.

**Total cases n=288**			58.2 (13.0)	171	217	

Key.

Appraisal with Joanna Briggs Institute critical appraisal tool for case-series.^32^ High quality = all criteria are met, Unclear quality = one or more criteria are unclear, Low quality = one or more criteria are not met.

Age in years.

SD = Standard Deviation.

JBI = Joanna Briggs Institute.

THA = Total hip arthroplasty.

SPECT/CT = Single photon emission computed tomography combined with computed tomography imaging.

CT = Computed tomography imaging.

MoM = Metal on metal.

MRI = Magnetic resonance imaging.

MARS = Metal artifact reduction sequence.

GTPS = Greater trochanteric pain syndrome.

CRP = C-reactive protein.

ESR = Erythrocyte sedimentation rate.

WBC = White blood cell.

NSAID = Nonsteroidal anti-inflammatory drug.

**Table 3:** The included case series were generally of high quality as assessed by the JBI case series assessment tool.

	Table 3: Critical appraisal										
Author, date	Result	1	2	3	4	5	6	7	8	9	10
Backer et al., 2020^11^	High quality	Y	Y	Y	Y	Y	Y	Y	Y	Y	Y
Berber et al., 2015^12^	High quality	Y	Y	Y	Y	Y	Y	Y	Y	Y	Y
Chalmers et al., 2017^13^	High quality	Y	Y	Y	Y	Y	Y	Y	Y	Y	Y
Dobrindt et al., 2015^14^	High quality	Y	Y	Y	Y	Y	Y	Y	Y	Y	NA
Erivan et al., 2019^15^	High quality	Y	Y	Y	Y	Y	Y	Y	Y	Y	Y
Hart et al., 2012^16^	High quality	Y	Y	Y	Y	Y	Y	Y	Y	Y	Y
Lahner et al., 2013^17^	Unclear quality	Y	Y	Y	UC	Y	Y	Y	Y	Y	Y
Tassinari et al., 2021^18^	High quality	Y	Y	Y	Y	Y	Y	Y	Y	Y	Y
Yun et al., 2021^19^	Unclear quality	Y	Y	Y	UC	Y	Y	Y	Y	Y	NA

Appraisal with the Joanna Briggs Institute Critical Appraisal tool: Checklist for Case-series.^32^

Y = Yes.

UC = Unclear.

NA = Not applicable.

**Table 4:** The most common cause of painful hip arthroplasty was attributed to prostatic loosening or infection, followed by iliopsoas impingement.

Table 4: Diagnoses		
Condition	Number	Percentage
Loosening, infection, or prosthesis failure	112	28.6 %
Iliopsoas impingement	82	21.0 %
Referred pain: Lumbar ♦	57	14.6 %
GTPS or trochanteric bursitis	51	13.0 %
Unknown	36	9.2 %
Pseudotumours	18	4.6 %
Heterotopic ossification.	8	2.0 %
Fracture∗	4	1.0 %
Referred pain: Knee ♦	3	0.8 %
Leg length discrepancy correction	3	0.8 %
Gluteal and piriformis myalgia	3	0.8 %
Muscle scarring	3	0.8 %
Instability without dislocation	3	0.8 %
Ischial tuberosity tendinitis	2	0.5 %
Neurological disorder ♦	2	0.5 %
Femoral acetabular impingement	2	0.5 %
Complex regional pain syndrome	1	0.3 %
Rhumatological condition ♦	1	0.3 %

Key.

♦ = Etiology outside the hip.

∗ = Fractures include, 1 contralateral metatarsal, 1 pubic bone, 1 greater trochanter, 1 ilio-ischiopubic ramus.

Study designs included prospective case-series,^11^^,^^12^^,^^17^ retrospective case-series,^13^, ^14^, ^15^^,^^18^^,^^19^ and a case-control study^16^ (Table 2). Four were imaging studies (109 hips),^11^^,^^12^^,^^14^^,^^16^ two discussed arthroscopic diagnosis and management (21 hips),^17^^,^^18^ one used a decision tree to reach a diagnosis (201 hips),^15^ one compared operative and non-operative management of iliopsoas impingement (49 hips),^13^ and one looked at revision surgery plus iliopsoas tenotomy in an iliopsoas impingement cohort (8 hips).^19^ A summary of the included studies presents critical appraisal result, study characteristics, population characteristics and findings (Table 2).

. A total of 388 painful hip arthroplasties were included in this analysis. There were 312 primary THA, 64 revision THA, and 12 resurfacing arthroplasties. The participants had an average age of 58.2yrs (SD 12.98). There were 217 females (56 %) and 171 males (44 %), Table 2.

The cause of pain remained unknown in 36 hips (9.2 %). The most common cause of persistent hip pain was loosening, infection or prosthesis failure, 112 cases (28.6 %). This combination of etiologies includes septic and aseptic loosening,^34^ low grade infection, component wear and failure of any prosthetic component. In some cases, multiple diagnoses were given for a single case such as “Early arthroplasty loosening, [with] low grade infection” ^11^. There were 82 reported cases (21 %) of iliopsoas impingement causing persistent pain. Causes that originated outside the hip accounted for 64 cases (16.4 %). These consisted of 57 lumbar referred pain (14.6 %), three knee referred pain (0.8 %), two neurological conditions (0.5 %), one rheumatological condition (0.3 %) and one metatarsal fracture (0.3 %). Greater trochanteric pain syndrome (GTPS) accounted for 51 of the cases (13 %) and, in line with the current definition, included specific etiologies of abductor deficiency, trochanteric bursitis, and abductor tendinopathy/tendinitis. A complete list of diagnoses is included in Table 2, Table 4.

Most of the participants (n = 236) had multiple imaging modalities to determine diagnosis. The most common imaging performed was plain radiograph, reported for all participants. Magnetic resonance imaging (MRI) was done on 86 participants,^11^^,^^12^^,^^15^^,^^16^ single-photon emission computed tomography (SPECT/CT) scan in 79, and computed tomography (CT) in 59. Ultrasonography was used in one study with 19 participants, Table 5.

**Table 5:** Xray followed by MRI was the most performed imaging.

Table 5: Imaging used in diagnostic process					
Author, date	X-ray	SPECT/CT	CT	MRI	Ultrasonography
Backer et al., 2020(11)	37	37		37	
Berber et al., 2015(12)	19	19		19	19
Chalmers et al., 2017(13)	49				
Dobrindt et al., 2015(14)	23	23			
Erivan et al.^a^, 2019(15)	201	NR	NR	NR	NR
Hart et al., 2012(16)	30		30	30	
Lahner et al., 2013(17)	5		5		
Tassinari et al., 2021(18)	16		16		
Yun et al., 2021(19)	8		8		
Totals	388	79	59	86	19

Key.

X-ray = Plain radiograph.

SPECT/CT = Single photon emission computed tomography/computed tomography.

CT = Computed tomography.

MRI = Magnetic resonance imaging.

NR = Number of cases this was used for was not reported.

a This study ^15^ reported the used of all these imaging modalities but lacked the details on which cases they were used for.

## 7 Discussion

This systematic review reports the investigation and diagnosis of painful etiologies in 388 hip arthroplasty patients. The included studies were generally of high quality, reported specific diagnoses and identified the diagnostic imaging modalities. However, they did not include other clinical signs and symptoms such as the amount or location of the pain which is likely important to the patient and a limitation of this review. Studies reported varying diagnostic methodology to systematically deduce the diagnosis. All but one study reported using two or more imaging techniques.^11^^,^^12^^,^^14^, ^15^, ^16^, ^17^, ^18^, ^19^ In most studies there was very limited reporting of clinical tests or other diagnostic techniques used in conjunction with the reported imaging modalities. Interestingly, despite extensive investigation, pain with an unknown cause was present in 9.2 % of cases.

This review confirms that loosening, infection and prosthetic failure is the most reported cause of post THA pain. These diagnoses were combined due to difficulty separating these data. For example, the largest study^15^ did not define loosening as septic or aseptic. Septic loosening is, by definition, caused by chronic infection at the implantation site ^34^. This proves to be problematic as septic loosening would require recording one case of infection, and one case of loosening. Additionally, where studies did not differentiate the type of loosening, it becomes impossible to accurately record if infection is present for analysis ^15^. Similarly, diagnoses of ‘cup loosening, [with] polyethylene wear’ includes both a prosthetic failure and loosening ^15^. To control for this potential bias of recording more than one etiology per case, we combined the etiologies that have overlapping causes and recorded each case only once, at the limitation of external validity. The authors justify this limitation by reiterating the aims of this review, which is to identify causes of pain, not conduct a prevalence study. All included studies reported screening for loosening, infection and prosthesis failure with blood tests and plain radiography. The presence of undetected bacterial cultures on orthopaedic devices is not unusual.^35^^,^^36^ A 1988 study found that 77 % of orthopaedic devices yielded signs of bacteria after being *in vivo* for two to 51 months.^35^ Another more recent study found one third of ‘aseptic’ hip and knee joint revisions contained causative organisms for joint infection.^36^ The high proportion infective diagnoses identified by imaging or arthroscopy^11^^,^^12^^,^^14^^,^^15^^,^^17^ may indicate that serum or synovial tests were not adequately performed in the diagnostic process. A more comprehensive diagnostic approach should be developed to adequately address these short comings.

Loosening and prosthesis failure were initially ruled out in all cases through plain radiography, and in some cases CT imaging.^16^^,^^19^ Despite these initial diagnostic tests, Loosening, infection and prosthesis failure were the underlying cause of pain in 28.6 % of cases. These finding support studies suggesting that undetected or dormant infection may be present in some orthopaedic devices.^36^^,^^37^ Because loosening, infection or prosthesis failure were reported together, the prevalence of these etiologies cannot be identified, thus no further conclusions can be drawn from this data.

Iliopsoas impingement (IPI) made up 21 % of cases in this review. This high presence is overrepresented in the present review due to the inclusion of three studies who only reported diagnoses of IPI (total = 73).^13^^,^^18^^,^^19^ The diagnostic techniques used to a reach diagnosis of IPI included physical examination with active straight leg raise, plain radiography±CT scan, and anesthetic injection into the iliopsoas tendon sheath relieving symptoms temporarily. Imaging focused on examining acetabular cup prominence and angular placement. These studies measured outcomes of surgical vs non-surgical intervention,^13^ success of arthroscopic iliopsoas tendon release,^18^ and iliopsoas tendon release while undergoing revision surgery.^18^ Excluding them from data analysis results in nine cases of IPI from the remaining 315 participants (2.8 %). This prevalence is closer to previous studies that reported prevalence's of 4.3 %^38^ and 3.9 %.^39^

Pain originating outside the hip region accounted for 64 cases (16.4 %). The most common diagnosis was referred pain from the lumbar spine, reported in 57 cases (14.6 %). Referred pain from the lumbar spine includes diagnoses of back pain with or without neuropathy. Only one study reported specifically screened for back pain during examination.^18^ They used CT to scan from the fourth lumbar vertebrae to the tibial plateau. This method let them exclude cases of referred pain in their population of IPI participants. This finding may indicate that more care should be taken to identify pain originating from the lumbar spine. Other studies that reported referred pain did not disclose how they reached these diagnoses. The remaining causes of hip pain such as referred from the knee, metabolic neuropathy, and non-union contralateral metatarsal fracture were rare in the present review. These were included in this study as they were the only attributable cause of pain reported for these cases. The authors' inclusion of metatarsal fracture could conceivably be because they considered it a secondary source of pain to the contralateral hip, via altered gait. In our view, however, these findings highlight the need to systematically evaluate, describe and exclude etiologies arising from outside the hip.

Greater trochanteric pain syndrome (GTPS) was present in 51 cases (13 %). In line with the current definition, GTPS included individual diagnoses of trochanteric bursitis, tendinitis, tendinopathy, and muscle tears of the hip abductor tendon, or myofascial trigger points over the trochanter.^40^ It has previously been established that MRI can detect asymptomatic tears in 13 % of THA participants.^41^ Asymptomatic abductor tendon tears pre-arthroplasty have been associated with poorer outcomes post-THA.^42^ It is possible pre-existing asymptomatic gluteal tendon tears become symptomatic after surgery and contribute to painful hip arthroplasty. Finally, trochanteric bursitis is rarely present in isolation and has been associated with Gluteus Medius and Gluteus Minimus tendinopathy in 90 % of cases.^43^ These findings support other studies that suggest GTPS may be the cause of persistent hip pain after THA in 12–22 % of participants.^44^^,^^45^ The link between painful hip arthroplasty identifies an area in need of further research.

Despite extensive investigation, the cause of persistent hip pain remained unknown in 36 cases (9.2 %). This inability to locate a source of pain highlights the difficulty clinicians face in reaching differential diagnoses. Referral to another specialty or a ‘wait and see’ approach was reported as the end point in some case.^11^^,^^12^^,^^14^ This high number of unknown diagnoses may be inflated in this review due to the inclusion of one study of metal-on-metal (MoM) arthroplasties that did not report pathologies other than pseudotumors.^16^ The selection and reporting biases in this study contribute to the limitations of this review. Excluding this study from the analysis results in 19 diagnoses (7.6 %) remaining unknown.

Pseudotumors, sometimes described as pseudocysts, were found in 18 cases (4.6 %) of which 17 were from MoM arthroplasties and one arthroplasty of unknown type. The predominant study that reported this etiology involved 12 hip resurfacing arthroplasties ^16^. There are established issues with MoM arthroplasties, as highlighted by published consensus statements. Discussions on these are beyond the scope of this review and readers are referred to the following research for clarification.^16^^,^^46^ However, This etiology should be considered as a cause of pain in patients with a MoM arthroplasty.^47^

Heterotopic ossification (HO) was detected as the cause of pain in eight cases (2 %). Most of these cases (n = 5) came from an imaging study using hybrid SPECT/CT (bone scintigraphy with SPECT/CT) scan to determine the cause of pain after uncemented arthroplasty.^14^ This study may have been more sensitive in picking up HO than other studies included in this review due to using hybrid SPECT/CT.^48^ It has been reported that bone scintigraphy is the most sensitive imaging modality for early detection of HO.^48^ Due to studies in this analysis excluding cases with known causes of pain, larger ossifications would have been excluded with plain radiography. The hybrid SPECT/CT used in this study may have detected small ossification that may have otherwise gone unnoticed. The size and location of the ossifications were not recorded in the three studies that reported these findings ^14, 15, 18^. A meta-analysis with over 6000 participants reported the incidence of HO after THA to be 30 %.^49^ Further research on hybrid SPECT/CT scan may indicate if this imaging modality should be used more commonly for painful hip arthroplasties.

Previously undetected fractures were found in four cases (1 %). Fractures were detected in a contralateral metatarsal, two in the greater trochanter, and one in the ilio-ischiopubic ramus. In the case of the metatarsal fracture the authors did not provide a hypothesis as to why this might cause contralateral hip pain. It may be theorised however, that the hip pain resulted from altered biomechanical load which occurred secondary to the fracture. Each fracture was not detected with plain radiography but were apparent with SPECT/CT imaging. This rare finding highlights the usefulness of plain radiography to rule out obvious fractures during preliminary screening.^50^ Despite this, unidentified fractures should be considered as a differential diagnosis in cases of persistent pain. The use of SPECT/CT or CT scan may be used to identify these etiologies when they do not appear on plain radiography.

There were several causes attributed to persistent pain that had a small prevalence in the reviewed population. Leg length (LL) discrepancy correction, Gluteal and Piriformis myalgia, abductor muscle scarring, and instability accounted for three cases each (0.8 %). Leg length discrepancy correction was described as a misplacement of the components while attempting to correct LL. Details on this were not elaborated on in the original study.^15^ Gluteal and Piriformis myalgia was the cause of pain in three cases (0.8 %). All of these were from the same study.^11^ This study used both MRI and SPECT/CT imaging to reach a diagnosis. Myalgia and muscle scaring at the greater trochanter possibly could have been included under GTPS however inadequate details provided in the studies prevented us from grouping them as such. Instability without dislocation was identified in one study and the etiology was not well defined.^15^ It is unclear if instability referred purely to subluxation of the joint, or if muscular weakness around the joint, an unstable arthroplasty coupling, cup malpositioning, or another etiology was present. Due to the limited explanation of this diagnosis and diagnostic methods, strong conclusions are difficult to establish.

Demographic differences included a female predominance suffering from persistent hip pain post arthroplasty (55.9 %). This finding was unusual because no individual study included or excluded participants based on sex. This was reflected in the populations of the two largest studies including a total of 201 (60.7 % female) ^15^ and 49 (53.1 % female) ^13^ participants. Several other studies have noted a female predominance in their participants.^21^ Research addressing this specific question is needed before conclusion can be drawn.

This review was limited by the inclusion of other types of hip arthroplasty and by poor reporting of diagnostic techniques and clinical features by the included studies. Specifically, despite excluding studies that only reported non-total hip arthroplasties, it was necessary to include studies that reported a non-hip arthroplasty along with total hip arthroplasty. This mixed data was unable to be differentiated in most cases.

## 8 Conclusion

This review identified the etiologies of persistent pain following hip arthroplasty and summarized diagnostic approaches. Some causes, such as loosening, infection, or prosthesis failure, may not be detected by standard tests and require further investigation. Less common sources of pain were also identified to aid differential diagnosis. While some pain originates within the hip, others arise from surrounding structures, with greater trochanteric pain syndrome (GTPS) potentially playing a larger role than previously recognized. The wide variation in clinical examination techniques, diagnostic methods, and imaging highlights the lack of consensus in evaluating persistent post-arthroplasty pain.

## CRediT authorship contribution statement

**Kael Hulin:** Investigation, Data curation, Writing – original draft, preparation, Formal analysis, Assoc Professor. **Angie Fearon:** Supervision, Methodology, Writing – review & editing, Professor. **Phil Newman:** Conceptualization, Methodology, Investigation, Supervision, Writing – review & editing.

## Patient consent

None required.

## Ethical clearance

None Required.

## Authors statements

The authors have no conflicts of interest to declare. No funding was received for this study. All authors have contributed to the manuscript and approved its final version for submission.

Authors: KH, AF, PN.

## Ethical statement

Not applicable-systematic review-no human participation.

## Guardian/patient consent

Not applicable-systematic review-no human participation.

## Funding statement

No funding has been received in relation to this publication.

## Conflict of interest statement from the authors

The authors of this manuscript certify that they have NO affiliations with or involvement in any organization or entity with any financial interest (such as honoraria; educational grants; participation in speakers' bureaus; membership, employment, consultancies, stock ownership, or other equity interest; and expert testimony or patent-licensing arrangements), or non-financial interest (such as personal or professional relationships, affiliations, knowledge or beliefs) in the subject matter or materials discussed in this manuscript.

## Figures and Tables

**Table:** 

	Population	Condition	Context
MeSH	o Arthroplasty, Replacement, Hip	o Chronic Pain o Pain, Intractable	o Clinical Decision-Making o Diagnosis, Differential
Free text	o Hip Prosthes∗ o Hip Replacement Arthroplast∗ o Total Hip Arthroplast∗ o Total Hip Replacement∗ o THA o THR	o Chronic Post operative Pain∗ o Chronic Post surgical Pain∗ o Chronic Post-operative Pain∗ o Chronic Post-surgical Pain∗ o Chronic Postoperative Pain∗ o Chronic Postsurgical Pain∗ o Persistent Postsurgical Pain∗ o Chronic Pain∗ o Intractable Pain∗ o Refractory Pain∗ o Painful THA o Painful total hip replacement o Painful hip replacement o Painful total hip arthroplasty o Painful hip arthroplasty	o Differential Diagnos∗ o Diagnos∗ and Examinations o Examinations and Diagnoses o Diagnos∗
Combine with “OR”	Yield = 30,779	Yield = 78,049	Yield = 1,261,861
Combine with “AND”, Yield = 192 (Articles published from 01/01/2012-06/05/2022 inclusive)			

**Table:** 

	Population	Condition	Context
MeSH	o Arthroplasty, Replacement, Hip	o Chronic Pain o Pain, Intractable	o Clinical Decision-Making o Diagnosis, Differential
Free text	o Hip Prosthes∗ o Hip Replacement Arthroplast∗ o Total Hip Arthroplast∗ o Total Hip Replacement∗ o THA o THR	o Chronic Post operative Pain∗ o Chronic Post surgical Pain∗ o Chronic Post-operative Pain∗ o Chronic Post-surgical Pain∗ o Chronic Postoperative Pain∗ o Chronic Postsurgical Pain∗ o Persistent Postsurgical Pain∗ o Chronic Pain∗ o Intractable Pain∗ o Refractory Pain∗ o Painful THA o Painful total hip replacement o Painful hip replacement o Painful total hip arthroplasty o Painful hip arthroplasty	o (Differential Diagnos∗ o Diagnos∗ and Examinations o Examinations and Diagnoses o Diagnos∗
Combine with “OR”	Yield = 15,722	Yield = 22,723	Yield = 436,868
Combine with “AND”, Yield = 23 (Articles published from 01/01/2012-06/05/2022 inclusive)			

**Table:** 

	Population	Condition	Context
Free text	o Hip Prosthes∗ o Hip Replacement Arthroplast∗ o Total Hip Arthroplast∗ o Total Hip Replacement∗ o THA o THR	o Chronic Post operative Pain∗ o Chronic Post surgical Pain∗ o Chronic Post-operative Pain∗ o Chronic Post-surgical Pain∗ o Chronic Postoperative Pain∗ o Chronic Postsurgical Pain∗ o Persistent Postsurgical Pain∗ o Chronic Pain∗ o Intractable Pain∗ o Refractory Pain∗ o Painful THA o Painful total hip replacement o Painful hip replacement o Painful total hip arthroplasty o Painful hip arthroplasty	o (Differential Diagnos∗ o Diagnos∗ and Examinations o Examinations and Diagnoses o Diagnos∗
Combine with “OR”	Yield = 30,323	Yield = 31,845	Yield = 1,779,588
Combine with “AND”, Yield = 30 (Articles published from 01/01/2012-06/05/2022 inclusive)			

## Appendix A

### Database search:

#### MEDLINE via Web of Science

(MH=(Arthroplasty, Replacement, Hip) OR TI=(Hip Prosthes∗) OR TI=(Hip Replacement Arthroplast∗) OR TI=(Total Hip Arthroplast∗) OR TI=(Total Hip Replacement∗) OR TI=(THA) OR TI=(THR) OR AB=(Hip Prosthes∗) OR AB=(Hip Replacement Arthroplast∗) OR AB=(Total Hip Arthroplast∗) OR AB=(Total Hip Replacement∗) OR AB=(THA) OR AB=(THR))

AND.

(MH=(Chronic Pain) OR MH=(Pain, Intractable) OR TI=(Chronic Post operative Pain∗) OR TI=(Chronic Post surgical Pain∗) OR TI=(Chronic Post-operative Pain∗) OR TI=(Chronic Post-surgical Pain∗) OR TI=(Chronic Postoperative Pain∗) OR TI=(Chronic Postsurgical Pain∗) OR TI=(Persistent Pain∗) OR TI=(Chronic Pain∗) OR TI=(Intractable Pain∗) OR TI=(Refractory Pain∗) OR AB=(Chronic Post operative Pain∗) OR AB=(Chronic Post surgical Pain∗) OR AB=(Chronic Post-operative Pain∗) OR AB=(Chronic Post-surgical Pain∗) OR AB=(Chronic Postoperative Pain∗) OR AB=(Chronic Postsurgical Pain∗) OR AB=(Persistent Pain∗) OR AB=(Chronic Pain∗) OR AB=(Intractable Pain∗) OR AB=(Refractory Pain∗) OR AB=(Painful THA) OR AB=(Painful total hip replacement) OR AB=(Painful hip replacement) OR AB=(Painful total hip arthroplasty) OR AB=(Painful hip arthroplasty) OR TI=(Painful THA) OR TI=(Painful total hip replacement) OR TI=(Painful hip replacement) OR TI=(Painful total hip arthroplasty) OR TI=(Painful hip arthroplasty))

AND.

(MH=(Clinical Decision-Making) OR MH=(Diagnosis, Differential) OR AB=(Differential Diagnos∗) OR AB=(Diagnos∗ and Examinations) OR AB=(Examinations and Diagnoses) OR AB=(Diagnos∗) OR TI=(Differential Diagnos∗) OR TI=(Diagnos∗ and Examinations) OR TI=(Examinations and Diagnoses) OR TI=(Diagnos∗))

#### CINHAL via EBSCOhost

(MH (“Arthroplasty, Replacement, Hip”) OR TI (“Hip Prosthes∗” OR “Hip Replacement Arthroplast∗” OR “Total Hip Arthroplast∗” OR “Total Hip Replacement∗” OR “THA” OR “THR”) OR AB (“Hip Prosthes∗” OR “Hip Replacement Arthroplast∗” OR “Total Hip Arthroplast∗” OR “Total Hip Replacement∗” OR “THA” OR “THR”))

AND.

(MH (“Chronic Pain” OR “Pain, Intractable”) OR TI (“Chronic Post operative Pain∗” OR “Chronic Post surgical Pain∗” OR “Chronic Post-operative Pain∗” OR “Chronic Post-surgical Pain∗” OR “Chronic Postoperative Pain∗” OR “Chronic Postsurgical Pain∗” OR “Persistent Postsurgical Pain∗” OR “Chronic Pain∗” OR “Intractable Pain∗” OR “Refractory Pain∗” OR “Painful THA” OR “Painful total hip replacement” OR “Painful hip replacement” OR “Painful total hip arthroplasty∗” OR “Painful hip arthroplasty∗”))) OR AB (“Chronic Post operative Pain∗” OR “Chronic Post surgical Pain∗” OR “Chronic Post-operative Pain∗” OR “Chronic Post-surgical Pain∗” OR “Chronic Postoperative Pain∗” OR “Chronic Postsurgical Pain∗” OR “Persistent Postsurgical Pain∗” OR “Chronic Pain∗” OR “Intractable Pain∗” OR “Refractory Pain∗” OR “Painful THA” OR “Painful total hip replacement” OR “Painful hip replacement” OR “Painful total hip arthroplasty∗” OR “Painful hip arthroplasty∗”))

AND.

(MH (“Clinical Decision-Making” OR “Diagnosis, Differential”) OR AB (“Differential Diagnos∗” OR “Diagnos∗ and Examinations” OR “Examinations and Diagnoses” OR “Diagnos∗”) OR (TI “Differential Diagnos∗” OR TI “Diagnos∗ and Examinations” OR TI “Examinations and Diagnoses” OR TI “Diagnos∗”))

Note: The Boolean operator ‘AND’ does not work in EBSCOhost. This string requires each section to be pasted into a different text box.

#### Scopus

(TITLE(“Hip Prosthes∗” OR “Hip Replacement Arthroplast∗” OR “Total Hip Arthroplast∗” OR “Total Hip Replacement∗” OR “THA” OR “THR”) OR ABS(“Hip Prosthes∗” OR “Hip Replacement Arthroplast∗” OR “Total Hip Arthroplast∗” OR “Total Hip Replacement∗” OR “THA” OR “THR”))

AND.

(TITLE (“Chronic Post operative Pain∗" OR “Chronic Post surgical Pain∗" OR “Chronic Post-operative Pain∗" OR “Chronic Post-surgical Pain∗" OR “Chronic Postoperative Pain∗" OR “Chronic Postsurgical Pain∗" OR “Persistent Postsurgical Pain∗" OR “Chronic Pain∗" OR “Intractable Pain∗" OR “Refractory Pain∗" OR “Painful THA” OR “Painful total hip replacement” OR “Painful hip replacement” OR “Painful total hip arthroplasty∗" OR “Painful hip arthroplasty∗") OR ABS (“Chronic Post operative Pain∗" OR “Chronic Post surgical Pain∗" OR “Chronic Post-operative Pain∗" OR “Chronic Post-surgical Pain∗" OR “Chronic Postoperative Pain∗" OR “Chronic Postsurgical Pain∗" OR “Persistent Postsurgical Pain∗" OR “Chronic Pain∗" OR “Intractable Pain∗" OR “Refractory Pain∗" OR “Painful THA” OR “Painful total hip replacement” OR “Painful hip replacement” OR “Painful total hip arthroplasty∗" OR “Painful hip arthroplasty∗")

AND.

(ABS(“Differential Diagnos∗” OR “Diagnos∗ and Examinations” OR “Examinations and Diagnoses” OR “Diagnos∗”) OR TITLE(“Differential Diagnos∗” OR “Diagnos∗ and Examinations” OR “Examinations and Diagnoses” OR “Diagnos∗”))

AND.

(LIMIT-TO (PUBYEAR, 2022) OR LIMIT-TO (PUBYEAR, 2021) OR LIMIT-TO (PUBYEAR, 2020) OR LIMIT-TO (PUBYEAR, 2019) OR LIMIT-TO (PUBYEAR, 2018) OR LIMIT-TO (PUBYEAR, 2017) OR LIMIT-TO (PUBYEAR, 2016) OR LIMIT-TO (PUBYEAR, 2015) OR LIMIT-TO (PUBYEAR, 2014) OR LIMIT-TO (PUBYEAR, 2013) OR LIMIT-TO (PUBYEAR, 2012)
